# Effectiveness of internet-delivered education and home exercise supported by behaviour change SMS on pain and function for people with knee osteoarthritis: a randomised controlled trial protocol

**DOI:** 10.1186/s12891-019-2714-x

**Published:** 2019-07-27

**Authors:** Rachel K. Nelligan, Rana S. Hinman, Jessica Kasza, Kim L. Bennell

**Affiliations:** 1 Centre for Health, Exercise and Sports Medicine, Department of Physiotherapy, School of Health Sciences, The University of Melbourne, Melbourne, VIC Australia; 2 School of Public Health and Preventive Medicine, Monash University, Melbourne, VIC Australia

**Keywords:** Knee osteoarthritis, Website, Internet, Exercise, SMS, Mobile phone, RCT, Trial, Adherence, OA

## Abstract

**Background:**

Knee osteoarthritis (OA) is a prevalent and chronic condition with no known cure. Exercise is advocated in all clinical guidelines due to its positive effects on symptoms. Despite this, exercise participation is often poor in people with knee OA with access to exercise treatments a known barrier. Internet-delivered exercise interventions have the potential to improve access to evidence-based exercise treatments and can benefit OA outcomes, although non-usage and low adherence potentially limit their effectiveness. Short message services (SMS) show promise in facilitating exercise adherence and may be one solution to improve adherence to internet-delivered exercise interventions. The combination of internet-delivered exercise and SMS adherence support has not been specifically evaluated in people with knee OA.

**Methods:**

This protocol reports a two-arm parallel-design, assessor- and participant-blinded randomised controlled trial. This trial is recruiting 206 people aged 45 years and older, with a clinical diagnosis of knee OA from the Australian-wide community. Eligible and consenting participants are enrolled and randomised to receive access to either i) ‘My Knee Education’, an education control website containing OA and exercise information only or ii) a combined intervention that includes a website, ‘My Knee Exercise’, containing the same educational information as the control, guidance to increase general physical activity, and the prescription of a 24-week self-directed home-based lower-limb strengthening program in addition to a 24-week behaviour change SMS exercise adherence program. Outcome measures are being collected at baseline and 24-weeks. Primary outcomes are self-reported knee pain and physical function. Secondary outcomes include another self-reported measure of knee pain, function in sport and recreation, quality-of-life, physical activity, self-efficacy, participant satisfaction and perceived global change.

**Discussion:**

This randomised controlled trial will provide evidence about the effectiveness of a combined intervention of internet-delivered OA and exercise education, physical activity guidance and prescription of a 24-week lower-limb strengthening exercise program supported by a behaviour change SMS program compared to internet delivered OA and exercise education alone.

**Trial registration:**

ACTRN12618001167257/13th July 2018.

**Electronic supplementary material:**

The online version of this article (10.1186/s12891-019-2714-x) contains supplementary material, which is available to authorized users.

## Background

Knee osteoarthritis (OA) is a chronic condition that affects the entire joint including cartilage, bone, ligament and muscle, with low grade inflammation typically present [1]. Knee OA is clinically diagnosed in people 45 years of age or over with activity related joint pain and morning joint stiffness lasting 30 min or less [2]. Symptoms include pain and impaired physical function which commonly leads to inactivity and reduced quality of life [3, 4]. Osteoarthritis is a leading contributor to global disability [5] with considerable economic costs and as such is a global public health problem [6]. With the number of people with symptomatic OA projected to steadily increase each year due to an ageing population and rising obesity [7], low-cost, scalable interventions that could be effective at a population level are needed to prevent an unmanageable increase in OA related health-care costs.

As knee OA is a chronic condition with no known cure, self-management and lifestyle modification are central to long-term symptom relief [8]. This includes exercise which has benefits on pain and physical functioning [9]. Despite this, exercise is underutilised in the management of knee OA [10–12]. A combination of factors may be responsible. General Practitioners (GPs), who commonly manage knee OA, report time pressures and lack of skill and/or insufficient knowledge to prescribe exercise to their patients [13, 14]. Exercise professionals, such as physiotherapists, who have the skills and knowledge are often not referred to [15, 16], and if consulted may not be equipped with sufficient training or skill to facilitate behaviour change in their patients to promote longer-term exercise adherence [17–20]. On an individual level, people with knee OA can lack the motivation [21], knowledge and confidence to commence, progress and adhere to self-directed exercise [22] and often have negative beliefs about the impact of exercise on their joints preventing initiation of exercise in the first place [23]. Challenges of access to exercise treatments also exist including associated costs and geographical location [24]. To overcome these barriers, innovative approaches to exercise education, prescription and adherence are needed.

Using technology to deliver exercise interventions may be one solution. Several self-directed OA targeted internet-delivered physical activity and/or exercise interventions have been described in the literature [25–28] with three evaluated in randomised controlled trials (RCTs) [25, 27, 29]. ‘First Step to Active Health’, a 12-week program with 4 exercise steps (cardiovascular, flexibility, upper and lower body strength, and balance) for users to incorporate over 12 weeks was compared to a nutrition control in adults with self-reported arthritis of any type [25]. Participants in the intervention group showed greater improvements in self-reported physical activity at 3 months, but not at 9 months, compared to control with similar gains in functional performance measures and arthritis self-efficacy. Measures of program usage and adherence were not reported. The ‘Join2Move’ program, a 9-week, 9-module physical activity program was compared to a wait-list control in adults with hip and/or knee OA [29]. At 3 months, participants receiving the intervention had significant improvements in self-reported physical function compared to the control, but this was not sustained at 12-months. Adherence to the intervention was problematic with 80% of participants completing the first and only 55% completing the second of nine modules. Finally, the 8-week ‘Help my Knees’ program was compared to standard in-person physiotherapy and a wait-list control in adults with knee OA [27]. The ‘Help My Knees’ program consisted of a progressive lower-limb strength and flexibility program and a walking program with access reminders sent via email if no program login was identified every 7 days. No significant between-group differences in pain or function were identified at 4 or 12 months. Exercise adherence was not reported, although engagement with the ‘Help My Knees’ program was overall low. Overall, the self-directed programs appeared safe with few adverse events reported [26, 27].

High rates of non-usage of internet-delivered exercise interventions and poor adherence to their recommendations is consistently reported [29–31]. Further research is needed to explore strategies to optimise adherence to these interventions and potentially enhance outcomes. A blended intervention where the internet resource is combined with face-to-face therapist contact is one option [32, 33]. However, this does not fully address access barriers to exercise treatments as patients are still required to travel to health care clinics. Another option may be the use of short message services (SMS) delivered using mobile phones. SMS can effectively promote adherence to a variety of health interventions including diabetes self-management and smoking cessation programs and health appointments [34, 35]. Specifically related to home-exercise adherence, the use of SMS shows promise in adults with knee OA [36], frozen shoulder [37] and healthy populations [38]. There are many benefits to using SMS to promote adherence to healthy behaviours. These include instantaneous communication and feedback [39], convenience, accessibility and cost-effectiveness [40] and user acceptability [41]. SMS programs underpinned by behaviour change theory appear to have greater effects [9]. The combination of an internet-delivered exercise intervention supported by a behaviour change SMS exercise adherence intervention has not been evaluated.

Therefore, we developed a 24-week combined intervention consisting of self-directed, internet-delivered OA education and exercise guidance, the ‘My Knee Exercise’ website, and a behaviour change SMS exercise adherence program. ‘My Knee Exercise’ is a website that includes knee OA and exercise educational information, guidance to increase general physical activity and describes a 24-week, progressive lower limb strengthening exercise program to be completed three times each week. The strengthening exercises have previously been found to reduce pain and improve physical function in people with knee OA when prescribed by a physiotherapist, remotely using video conferencing [42].

The behaviour change SMS exercise adherence program was developed applying the Behaviour Change Wheel (BCW), a synthesis of 19 models of behaviour which can be applied to inform behaviour change intervention design [43, 44]. The BCW uses the Capability, Opportunity, M motivation model of behaviour (COM-B) and the Theoretical Domains Framework (TDF) [45] to guide selection of barriers/facilitators of a behaviour. The COM- B model includes six categories to describe behaviour; i) physical capability (e.g physical skill); ii) psychological capability (e.g knowledge and psychological skill); iii) physical opportunity (e.g. the environment such as time/resources); iv) social opportunity; v) reflective motivation (e.g. self-conscious intentions and beliefs); and vi) automatic motivation (e.g. emotional reactions, desires, impulses). The TDF includes 14 additional domains that link to COM-B categories [19]. These domains can be used to understand a behaviour in greater detail if needed. Once barriers/facilitators of the behaviour are identified and linked to COM-B/TDF the BCW then suggests intervention functions (e.g. education or training) which may bring about change and guides selection of behaviour change techniques (BCTs) from the Behaviour Change Technique Taxonomy (BCTTv1) [46] to use in the intervention.

Development of the SMS program is reported elsewhere (manuscript provisionally accepted). In brief, the program is a 24-week automated program that aims to facilitate adherence to the strengthening exercise program, 3 times weekly. The program prompts weekly self-reporting of exercise sessions completed in the previous week, addresses key barriers and facilitators to exercise adherence in knee OA [23], and provides BCT suggestions to address these barriers/facilitators. One hundred and ninety-eight SMS were constructed to form the SMS program library with SMS wording input from 16 people including the authors (academics, clinical physiotherapists and a person with knee OA). The finalised SMS library was provided to an external company who developed the automated SMS program.

The primary aim of this pragmatic randomised controlled trial (RCT) is to evaluate the effect of this combined intervention consisting of the “My Knee Exercise” website and SMS adherence support compared to a control website that contains standard OA and exercise educational information alone, like what is already currently available on the internet. Our primary hypothesis is that participants who receive “My Knee Exercise” and the SMS support will have greater improvements in pain and function compared to those who receive the education control at 24-weeks. Our secondary hypothesis is that improvements in other outcomes such as other measures of pain, health-related quality-of-life, physical activity, self-efficacy, global change and satisfaction will be greater in the combined intervention compared to the education control group.

## Methods/design

### Trial design

**Fig. 1:**
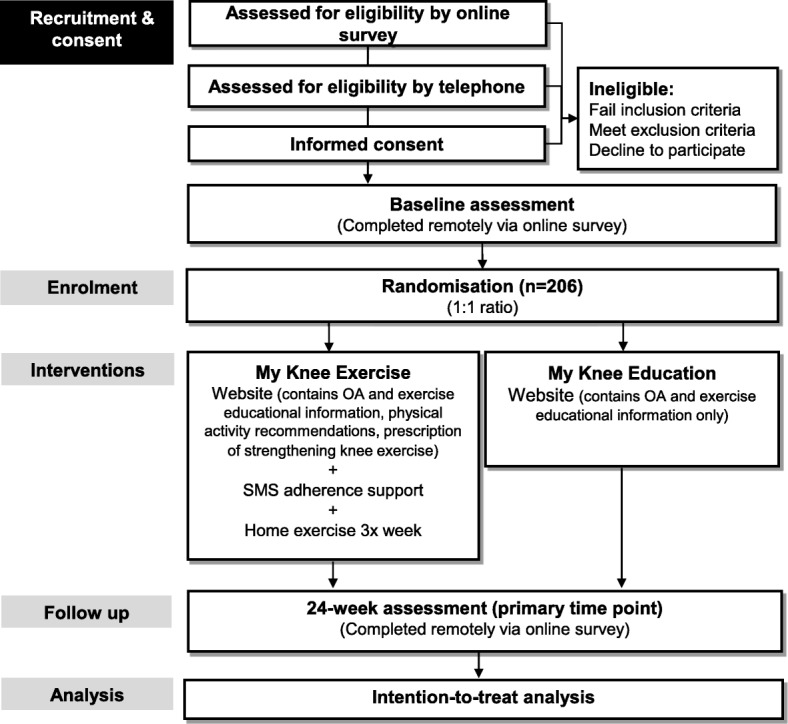
Flow diagram of trial procedures

This protocol reports a pragmatic, 2-arm, parallel-design, assessor- and participant- blinded randomized controlled trial and complies with SPIRIT guidelines [47]. Reporting will comply with CONSORT [48–51] and TIDieR guidelines [47]. Trial phases are outlined in Fig. 1.

### Participants

Informed by a sample size calculation, 206 participants are being recruited from the Australia-wide community via social media, internet-based newspapers and from our volunteer study database. Participants are ≥45 years of age and have a clinical diagnosis of knee OA [8]. Table 1 lists the full eligibility criteria. Ethics approval has been obtained from the Human Research Ethics Committee of University of Melbourne (HREC No. 1851085.1).

**Table 1:** Trial inclusion/exclusion criteria

Inclusion criteria	Exclusion criteria
Meet the National Institute for Health and Care Excellence (NICE) osteoarthritis clinical criteria [8]: - aged 45 years and over - activity related knee joint pain - morning stiffness ≤30 min	Booked for knee joint replacement surgery;
History of knee pain on most days for ≥3 months	Have had a knee joint replacement in the most painful knee
Overall average knee pain in past week self-rated as ≥4 out of 10 on an 11-point numeric rating scale (NRS)	Have participated in a structured knee exercise program within the past 6 months
Able to give informed consent and to participate fully in the interventions and assessment procedures	Self-reported diagnosis of rheumatoid arthritis or other inflammatory arthritis
Have a mobile phone with text message functioning and be willing to use it during the study to receive and send text messages	Have had a fall within the last 12 months and do not receive clearance from a General Practitioner to participate in an unsupervised home exercise program
Have a home Internet connection and a device that enables access to the Internet.	Are housebound requiring assistance from another person to leave the house in the previous month and do not receive medical clearance from a General Practitioner to participate in an unsupervised home exercise program
	Fail pre-exercise screening [73] and do not receive medical clearance from a General Practitioner to participate in an unsupervised home exercise program
	Unable to speak or read English

### Procedures

Baseline and 24-week assessments are being collected remotely via password protected online surveys using RedCap™ software. Data will be deidentified and downloaded into Microsoft® Office Excel software and stored in password protected computer files on the university server. All authors will have access to the final trial dataset. The most symptomatic knee at the time of enrolment is the focus of evaluation. In cases where both knees are equally symptomatic and eligible, the right knee is deemed the study knee. All participants provide informed consent to participate via online consent forms, prior to completing baseline assessments.

### Randomization and blinding

On completion of baseline assessment, participants are randomised into one of two groups i) My Knee Exercise and SMS support; or ii) My Knee Education. Computer-generated randomisation has been prepared by our study biostatistician (JK) in permuted blocks of sizes 6 to 12. To conceal allocation, the randomisation schedule is accessed via a password-protected, computer program by a researcher not involved in participant recruitment scheduling or assessment. The person who determines if a potential participant is eligible for inclusion in the trial is unaware, when this decision is made, to which group the participant will be allocated. Participants are blinded to study groups, informed at the time of recruitment that the study will investigate and compare digital resources/websites aimed at helping people self-manage their knee pain and that this may include exercise and mobile phone text message contact. Data will be analysed with blinding to group allocation. As outcomes are self-reported measures and participants are blinded this constitutes assessor blinding.

### Interventions

#### My Knee Exercise website and SMS support (Intervention)

Participants in this group receive access to the ‘My Knee Exercise’ website and a 24-week behaviour change SMS program. After randomisation, participants receive an email from the study co-ordinator, which contains details of how to access the website (the URL and their unique username and password). Participants are asked to access the website at home, on their own device within 7 days and are informed they can view the website as often as they require. Participants also receive a welcome SMS prompting them to access the website and start the prescribed knee strengthening exercise within the coming 7 days.

##### My Knee Exercise website

The website contains a home page and four sections that provide educational information, guidance to increase general physical activity, a 24-week home-based lower limb strengthening exercise program and resources to support exercise (e.g. printable logbooks). The website was constructed by the authors who received website development training from a web-designer. The website was developed in accordance with recommendations outlined by The Health on the Net Foundation’s Code of Conduct [52]. This code provides an ethical standard for the reliability of internet-delivered medical and health information. Three people with knee OA provided input on the website prototype regarding design, content and usability which informed the finalised website. The literacy demands of all written material in the website was evaluated with recommended [53] and previously used [54, 55] online readability software (Readable.io, Added Bytes, Ltd., Sussex, UK) as suitable for individuals with a 5th-grade reading ability. This is in accordance with the recommended reading level for consumer healthcare information of no greater than 6th–8th grade [56, 57]. Material within the website was specifically developed for this study. The website is not tailored to each participant. All participants receive the same standardised website. Website sections are discussed in detail below.

Home Page: The website home page includes a video tutorial called “Start here.” This tutorial explains and models use of each section of the website, encourages participants to start the strengthening exercise as soon as possible and explains the SMS exercise support. The home page also contains links to the four sections of the website plus links to ‘contact us’ and ‘about us’ information.

My Knee Education: This section contains written information about living with knee OA, knee OA treatments, exercise as treatment for knee OA, recommended exercise for knee OA and managing exercise pain. Written information is supported by video interviews of people with knee OA and OA experts. Participants are directed to view the written and audio-visual material prior to commencing the strengthening exercise program prescribed within the ‘My Knee Strength’ section of the website. Participants can download copies of all written information.

My Knee Strength: This section describes a 24-week, self-directed, home-based, lower limb strengthening exercise program. Over 24-weeks, participants are instructed to complete three, eight-week exercise programs, completed in succession. The exercise protocols were chosen based on those demonstrated to reduce pain and improve physical function in people with knee OA when prescribed by a physiotherapist [42, 58]. Participants receive a SMS at week one to prompt commencement of exercise program one and a SMS at week eight and week 16 to encourage use of the website and progression to exercise program two and three, respectively. Program one and two contain five exercises and program three contains six exercises. Exercises in each program target the hip, knee and ankle, and include for example seated knee extension, walk squats, hip abduction and calf raises. Two to three new exercises replace other exercises in each program to add variety and increase the exercise challenge. Exercise dosage, in accordance with recommendations for muscle hypertrophy [59], is: 10 repetitions; three sets with 2 min rest between each set; three times a week; at an exercise intensity of hard [4, 5] in program one and very hard [6, 7] in program two and three, as rated on an 11-point scale of Rated Perceived Exertion (RPE) for strength training [60]. Intensity is increased for each exercise by adding ankle weights and/or changing body position. In line with a pragmatic approach, participants are encouraged to purchase their own ankle weights to progress their exercises throughout the 24-week intervention. Each exercise session is estimated to be 20–30 min duration. Written instructions are provided for each exercise as well as photographic and video demonstrations. Participants can download and print a copy of each exercise program. Downloadable exercise logbooks for each program are also provided. Participants are encouraged, but not mandated, to use the log books to record their weekly exercise practice. ‘My Knee Strength’ also contains suggestions of what and where to purchase exercise equipment to progress the exercises, tips for starting and sticking to exercise, and provides details about the SMS exercise adherence program.

My Knee Physical Activity: This section contains information and guidance to assist participants to increase their general physical activity over the 24-week intervention period. Information includes why and how to increase general physical activity, how to track and safely increase daily steps, activity pacing and how to make a physical activity plan. A physical activity plan and a physical activity log book are available in downloadable PDF format. This section also includes video interviews of people with knee OA discussing their experience increasing activity in the presence of knee pain.

My Knee Tools: This section synthesises downloadable resources from the other three sections of the website including a physical activity plan template, strengthening program one, two and three logbooks; and a physical activity logbook. Exercise equipment suggestions, tips for starting and sticking to exercise and video interviews of people with knee OA discussing their experience with knee exercise and increasing activity with knee pain are also included.

##### SMS exercise adherence support

The development of the SMS program is reported elsewhere (manuscript provisionally accepted). In summary, over the 24-week intervention period participants receive two to five SMS weekly. Message frequency declines over time to reduce user burden, as recommended [61]. SMS length ranges from 105 to 420 characters and are personalised by using the participant’s first name. The SMS program is mentioned in the website, but they are not linked. The start of the automated SMS program is triggered by the study co-ordinator at the time of participant enrolment. The study co-ordinator enters, into an online platform, a start date for when the first automated SMS will be sent. To allow participants enough time to access the website and start their strengthening exercise, the program start date is the next Monday that is 5 days or more after enrolment. On the start date and proceeding Mondays (weekly between weeks 1–8 and fortnightly between weeks 9–24), users receive a SMS asking them to self-report the number of knee strengthening exercise sessions completed in the previous week. If users self-report ≤2 exercise session/week (classified as low adherence) they then receive a SMS that prompts them to select a barrier from a prespecified list (forgot, too tired, knee hurts so can’t exercise, worried exercise is causing pain, exercise isn’t helping, boring, lack of time, life stress, and none above apply to me) which best explains why they were unable to complete their exercises three times in the previous week, as recommended in the website. The user’s barrier selection triggers a SMS that contains a BCT suggestion related to the selected barrier. Users who report ≥3 exercise session/week (classified as adherent) receive a SMS that encourages continued completion of the exercises ≥3/week. Users also receive regular SMS (twice weekly initially and reducing to once fortnightly by 24-weeks) which contain BCT suggestions to facilitate completion of the exercises ≥3/week, irrespective of their self-reported adherence. Program automation prevents the same BCT SMS being sent for example if the same barrier is selected more than once. Additional files 1 and 2 list the barriers and facilitators used in the SMS program and the BCTs selected to address them. To further enhance engagement, participants are sent special occasion messages (e.g. birthday, Christmas).

#### My Knee Education (control)

Participants in the control condition are provided with a URL to access ‘My Knee Education’, a website containing only the educational information provided in the intervention within the ‘My Knee Education’ section with any reference to the intervention exercise program removed and replaced with general exercise and physical activity recommendations, like those available in current Internet-based Australian OA consumers resources. The control site does not contain an exercise program. All material within the website has been specifically developed for this study. After randomisation, participants receive an email, from the study co-ordinator, containing details of how to access the website (the URL and their unique username and password). Participants are asked to access the website at home, on their own device within 7 days, read the educational information provided and implement any exercise and physical activity recommendations as they see fit. Participants are instructed that they can view the website as often as they require. At the same time participants receive their access email they also receive a welcome SMS prompting them to access the website.

### Outcomes

Outcome measures are self-reported. The primary time point is 24-weeks after baseline. Table 2 summarises all measures captured.

**Table 2:**
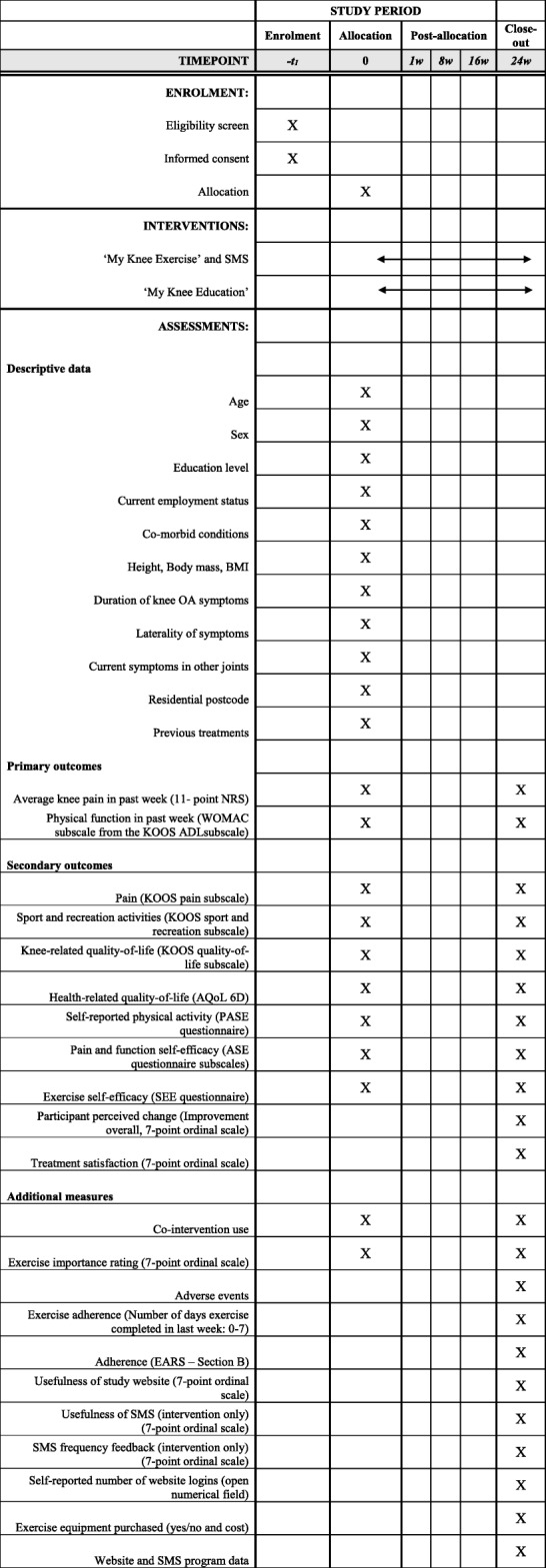
Schedule of enrolment, intervention, and assessments

*BMI* Body Mass Index, *EARS* Exercise Adherence Rating Scale, *NRS* numeric rating scale, *WOMAC* The Western Ontario and McMaster Universities Osteoarthritis Index, *KOOS* Knee Osteoarthritis Outcome Score, *ADL* Activity of Daily Living, *AQoL2* Assessment of Quality of Life Instrument, *PASE* Physical Activity scale for the elderly, *ASE* Arthritis Self Efficacy scale, *SEE* Self- efficacy for exercise scale, *SMS* Short Message Service

#### Descriptive and other measures

Age, sex, education level, current employment status, co-morbid conditions, duration of knee OA symptoms, laterality of symptoms, previous treatments and medications, problems in other joints, residential postcode and self-reported height and body mass are obtained at baseline. Body mass index is calculated using height and weight measures.

#### Primary outcomes

Primary outcomes are collected at baseline and 24-weeks.

*Overall knee pain*: Average overall knee pain in the past week is self-assessed using a 11-point numeric rating scale (NRS) [62] with terminal descriptors of ‘no pain’ (score 0) and ‘extreme pain’ (score 10).

*Physical function*: Limitations with physical functioning over the past week are measured by extracting the Physical Function subscale of the Western Ontario and McMaster Universities Osteoarthritis Index (WOMAC) from the Activity of Daily Living subscale of the Knee Injury and Osteoarthritis Outcome Score (KOOS) [63], which contains the WOMAC questions. It is a self-report measure with established validity, reliability and responsiveness in knee OA [64]. The physical function subscale contains 17 questions with response captured on a 5-point scale with terminal descriptors 0 = “none” to 4 = “extreme”. Total score ranges from 0 to 68 with lower scores indicating worse functioning.

#### Secondary outcome measures

Self-reported secondary outcomes are measured at baseline and 24 weeks, unless indicated otherwise.

*Pain, function in sport and recreation and knee-related quality of life in the last week:* Additional subscales of the Knee Injury and Osteoarthritis Outcome Score (KOOS) are being measured [63] including i) pain (9-items), ii) function in sport and recreation (5-items) and iii) knee-related quality of life quality of life (4-items). Responses are provided on a 5-point scale. Scores will be calculated for each subscale and range from 0 to 100 with 0 indicating worst possible symptoms.

*Health-related quality of life:* The AQoL questionnaire (version AQoL-II) [65] is measuring health related quality of life. This is a 20-item measure with responses provided on a 5-point scale. Scores range from − 0.04 to 1.00 with 1 indicating full health-related quality of life.

*Physical activity*: The Physical Activity Scale for the Elderly [66] is being used to assess physical activity, over the previous week. This is a 10-item measure which collects responses for the frequency, duration, and intensity level of a range of activities typically chosen by older adults. Scores range 0 to > 400 with higher scores indicating greater levels of physical activity.

*Self-efficacy:* The Arthritis Self-Efficacy Scale [67] is being used to measure arthritis specific self-efficacy. Two subscales are being collected, self-efficacy for managing pain (5-items) and physical function self-efficacy (9-items) with responses provided on a 10-point scale. Scores for each subscale range from 1 to 10 with higher scores indicating greater self-efficacy.

*Exercise self-efficacy:* The Self-efficacy For Exercise (SEE) Scale is measuring exercise self-efficacy. This is a 9-item measure with responses provided on a 11-point scale. Scores range from 0 to 90 with higher scores indicates higher self-efficacy for exercise.

*Participant-perceived change overall due to treatment (24 week only)*: Participants rate their perceived change in their condition overall, since baseline, on a 7-point scale with terminal descriptors of 1 = “much worse” to 7 = “much better”. Scores of 6 (“moderately better”) and above will be considered to indicate improvement [68].

*Overall satisfaction (24 week only):* Participants rate their overall satisfaction with the digital resource(s) they accessed as part of the study on a 7-point scale with terminal descriptors of 1 = “very dissatisfied” to 7 = “very satisfied”. Scores range from 1 to 7 with higher scores indicating greater satisfaction.

#### Process measures

A range of process measures are assessed at 24-weeks, unless indicated otherwise.

*Exercise importance (baseline and 24-weeks)*: Participants will be asked the question “How important is it to you to do regular exercise to manage your knee condition?” Responses are collected on a 7-point scale with terminal descriptors 1 = ‘not at all important’ and 7 = ‘extremely important’. Scores range from 1 to 7, with higher scores indicating higher importance.

*Self-reported number of exercise days in the previous week:* Participants are asked “In the past week, how many days did you participate in exercise for your knee condition?” Scores range from 0 to 7.

*Adherence to prescribed home exercise:* Exercise Adherence Rating Scale (EARS) Section B is being used to measure adherence to prescribed home exercise [69]. This is a 6-item measure. Responses are collected on a 5-point scale. Scores range from 0 to 24 with higher score indicating better adherence.

*Perceived usefulness of study website*: Participants are asked to report their level of agreement with the statement “I thought the website I accessed as part of the study was useful in helping me manage my painful knee”. Responses will be collected on a 7-point scale with terminal descriptors 1 = ‘strongly disagree’ and 2 = ‘strongly agree’. Scores range from 1 to 7 with higher scores indicating higher perceived usefulness.

*Perceived usefulness of SMS exercise adherence support (intervention only)*: Participants are asked to report their level of agreement with the statement “I thought the mobile phone text messages I received were useful in helping me manage my painful knee”. Responses will be collected on a 7-point scale with terminal descriptors 1 = ‘strongly disagree’ and 2 = ‘strongly agree’. Scores range from 1 to 7 with higher scores indicating higher perceived usefulness.

*SMS frequency feedback (intervention only)*: Participants agreement with the statement “The number of text messages I received over the past 6 months was the right amount for me.” Responses will be collected on a 7-point scale with terminal descriptors 1 = ‘strongly disagree’ and 2 = ‘strongly agree’. Scores range from 1 to 7 with higher scores indicating higher agreement. Participants who select ≥3 will be asked ‘Did you receive too many or too few?’ with response collected via 1 = ‘too few’ or 2 = ‘too many’.

*Website access:* Participants are asked to estimate how many times they accessed their study website in: i) the first month of the study, and ii) month five of the study. Responses are collected in an open numerical field box.

*Exercise equipment purchased:* Participants will be asked if they purchased any exercise equipment to help them with their knee exercise. Participants who select ‘yes’ will be asked to estimate the cost of the equipment purchased in an open numerical field box.

*Website and SMS program data:* Interaction data will be collected from both websites and the SMS program such as website logins and SMS program ‘opt-outs’.

*Adverse events*: Any problem that participants believe was caused by the information received as part of the study and required them to seek treatment/take medications, and/or interfered with function for two or more days are recorded via questionnaire.

*Co-interventions:* Medications and other treatments for knee OA are recorded at 24 weeks using a custom-developed survey. This survey records the frequency of use of a range of pain and arthritis medications and co-interventions over the past 6 months.

### Data analysis, monitoring and auditing

#### Sample size calculation

The sample size is based on detecting an effect size of 0.40 for the two primary outcomes of pain and WOMAC physical function. According to Cohen [70], an effect size of 0.40 represents a small to moderate difference between groups. This effect size was chosen based on mean effect sizes for exercise treatment in people with knee osteoarthritis. A meta-analysis of 44 clinical trials investigating land-based exercise showed effect sizes of 0.49 for pain and 0.52 for physical function [9]. As the current trial includes unsupervised exercise, which we believe may result in smaller effects, we powered the trial to detect a more conservative effect size of 0.40 for both primary outcomes. Thus, to achieve 80% power and 0.05 two-sided significance level, with a correlation between pre- and post-measurements of 0.35 for pain and function, and accounting for 15% loss to follow up [42, 58], we require 103 participants per arm, for a total of 206 participants. Assuming between-participant standard deviations of 2.3 for pain and 11.7 for WOMAC physical function based on our previous research [58] and a pre-post correlation of 0.35, this sample size of 206 participants will provide us with > 99% power to detect a minimal clinically important difference in pain of 1.8 units [71] and 95% power to detect a minimal clinically important difference in function of 6 WOMAC units [72].

#### Data analysis

Analyses comparing the two groups will be performed by the statistician in a blinded fashion using all available data from all randomised participants according to the intention-to-treat principle. Should the amount of missing data for an outcome be greater than 5%, multiple imputation will be conducted, and the method reported. The primary analysis will analyse the multiply imputed datasets. Demographic and baseline characteristics of participants will be summarised as appropriate (means and standard deviations for continuous variables that appear to be distributed approximately symmetrically, medians and interquartile ranges for other continuous variables, counts and percentages for categorical variables) and will be inspected to assess baseline comparability of treatment groups. For continuous outcomes, differences will be compared between groups in either change (baseline minus follow up) or at 24 weeks using linear regression models adjusted for baseline levels of these outcomes. Model assumptions will be assessed using standard diagnostic plots. We will also calculate the proportion of participants in each group that show an improvement that reaches or exceeds the minimal clinically important difference in NRS pain (≥1.8 units) and in WOMAC function (≥6 units). For this and other binary outcomes, differences between groups will be compared using relative risks, calculated from logistic regression models.

#### Monitoring

Monthly meetings are held between the trial coordinator and lead investigators to review recruitment, trial progress, monitor adverse events and any other issues relating to the trial.

### Dissemination plans

Trial findings will be disseminated at conferences, published in a peer-reviewed journal and a lay summary of findings provided to all participants. Findings will also be dissemination through research networks including the Centre for Health, Exercise and Sports Medicine, and the National Health and Medical Research Council Centre for Research Excellence in Translational Research in Musculoskeletal Pain. We anticipate making the ‘My Knee Exercise’ website available for the general population to access.

## Discussion

This RCT is investigating if a digitally delivered intervention that combines "My Knee Exercise’, a website containing knee OA and exercise education, guidance to increase general physical activity, and prescribes a 24-week home-based lower limb strengthening program with a behaviour change SMS exercise adherence program can improve knee pain and physical function at 24-weeks when compared to OA and exercise education alone. If effective, this combined intervention will be an easily scalable intervention with the potential to increase access to exercise programs for the treatment of knee OA. This intervention could be easily disseminated by primary care doctors and health professionals as a first- line intervention in a stepped care approach, provided by health insurers for their members and/or linked to currently available Internet delivered OA consumer resources.

## Additional files

Additional file 1 Exercise barriers and the BCTs which were converted into individual SMS to address each barrier. Demonstrates the application of the BCW framework using COM-B categories, TDF domains and intervention functions. (PDF 63 kb)

Additional file 2 Exercise facilitators and the BCTs which were converted into individual SMS to address each facilitator Demonstrates the application of the BCW framework using COM-B categories, TDF domains and intervention functions. (PDF 35 kb)

## Data Availability

The SMS programs behaviour change message library will be available by request on a case-by-case basis at the discretion of the corresponding author.

## Abbreviations

BCT: Behaviour change technique
BCW: Behaviour Change Wheel
COM-B: Capability, Opportunity and Motivation model of behaviour
OA: Osteoarthritis
SMS: Short Messaging Service
TDF: Theoretical Domains Framework

## References

[1] Jones G (2016). What’s new in osteoarthritis pathogenesis?. Intern Med J.

[2] NICE (2014). National Institute for health and clinical excellence: guidance. Osteoarthritis: Care and Management in Adults.

[3] Dieppe P, Cushnaghan J, Tucker M, Browning S, Shepstone L (2000). The Bristol ‘OA500 study’: progression and impact of the disease after 8 years. Osteoarthr Cartil.

[4] Sharma L, Cahue S, Song J, Hayes K, Pai YC, Dunlop D (2003). Physical functioning over three years in knee osteoarthritis: role of psychosocial, local mechanical, and neuromuscular factors. Arthritis Rheum.

[5] Cross M, Smith E, Hoy D, Nolte S, Ackerman I, Fransen M (2014). The global burden of hip and knee osteoarthritis: estimates from the global burden of disease 2010 study. Ann Rheum Dis.

[6] Salmon JH, Rat AC, Sellam J, Michel M, Eschard JP, Guillemin F (2016). Economic impact of lower-limb osteoarthritis worldwide: a systematic review of cost-of-illness studies. Osteoarthr Cartil.

[7] Australia A. Painful realities: the economic impact of arthritis in Australia in 2007. Sydney; 2007.

[8] Excellence NIfHaC. Osteoarthritis: Care and Management: Clinical Guidlelines. London: National Institute for Health and Care Excellence (UK) Copyright (c) National Clinical Guideline Centre; 2014.

[9] Fransen M, McConnell S, Harmer AR, Van der Esch M, Simic M, Bennell KL (2015). Exercise for osteoarthritis of the knee: a Cochrane systematic review. Br J Sports Med.

[10] Hinman RS, Nicolson PJ, Dobson FL, Bennell KL (2015). Use of nondrug, nonoperative interventions by community-dwelling people with hip and knee osteoarthritis. Arthritis Care Res.

[11] Hunter DJ (2010). Quality of osteoarthritis care for community-dwelling older adults. Clin Geriatr Med.

[12] Basedow M, Williams H, Shanahan EM, Runciman WB, Esterman A (2015). Australian GP management of osteoarthritis following the release of the RACGP guideline for the non-surgical management of hip and knee osteoarthritis. BMC Res Notes.

[13] Cottrell E, Roddy E, Rathod T, Porcheret M, Foster NE (2016). What influences general practitioners’ use of exercise for patients with chronic knee pain? Results from a national survey. BMC Fam Pract.

[14] Thorlene E, Nelligan RK, Setchell J, Atkins L, Bennell BL (2018). General practitioners’ views on managing knee osteoarthritis: A thematic analysis of factors influencing clinical practice guideline implementation in primary care. BMC Rheumatol.

[15] Foster NE, Hartvigsen J, Croft PR (2012). Taking responsibility for the early assessment and treatment of patients with musculoskeletal pain: a review and critical analysis. Arthritis Res Ther.

[16] Brand CA, Harrison C, Tropea J, Hinman RS, Britt H, Bennell K (2014). Management of osteoarthritis in general practice in Australia. Arthritis Care Res.

[17] Holden MA, Nicholls EE, Young J, Hay EM, Foster NE (2009). UK-based physical therapists’ attitudes and beliefs regarding exercise and knee osteoarthritis: findings from a mixed-methods study. Arthritis Rheum.

[18] Mudge S, Stretton C, Kayes N (2014). Are physiotherapists comfortable with person-centred practice? An autoethnographic insight. Disabil Rehabil.

[19] Lawford BJ, Delany C, Bennell KL, Bills C, Gale J, Hinman RS (2018). Training physical therapists in person-centered practice for people with osteoarthritis: a qualitative case study. Arthritis Care Res.

[20] Rhodes RE, Fiala B (2009). Building motivation and sustainability into the prescription and recommendations for physical activity and exercise therapy: the evidence. Physiother Theory Pract.

[21] Hurley M, Dickson K, Hallett R, Grant R, Hauari H, Walsh N (2018). Exercise interventions and patient beliefs for people with hip, knee or hip and knee osteoarthritis: a mixed methods review. Cochrane Database Syst Rev.

[22] Ravaud P, Giraudeau B, Logeart I, Larguier JS, Rolland D, Treves R (2004). Management of osteoarthritis (OA) with an unsupervised home based exercise programme and/or patient administered assessment tools. A cluster randomised controlled trial with a 2x2 factorial design. Ann Rheum Dis.

[23] Dobson F, Bennell KL, French SD, Nicolson PJ, Klaasman RN, Holden MA (2016). Barriers and facilitators to exercise participation in people with hip and/or knee osteoarthritis: synthesis of the literature using behavior change theory. Am J Phys Med Rehabil.

[24] Hinman RS, Nelligan RK, Bennell KL, Delany C (2017). “Sounds a bit crazy, but it was almost more personal:” a qualitative study of patient and clinician experiences of physical therapist-prescribed exercise for knee osteoarthritis via skype. Arthritis Care Res.

[25] Wilcox S, McClenaghan B, Sharpe PA, Baruth M, Hootman JM, Leith K (2015). The steps to health randomized trial for arthritis: a self-directed exercise versus nutrition control program. Am J Prev Med.

[26] Bossen D, Veenhof C, Dekker J, De Bakker D (2012). The design and feasability of a web-based physical activity program for patients with osteoarthritis of hip or knee. J Sci Med Sport.

[27] Brooks MA, Beaulieu JE, Severson HH, Wille CM, Cooper D, Gau JM (2014). Web-based therapeutic exercise resource center as a treatment for knee osteoarthritis: a prospective cohort pilot study. BMC Musculoskelet Disord.

[28] Dahlberg LE, Grahn D, Dahlberg JE, Dodge GR, Thorstensson C. Joint academy-an innovative internet-based platform for the management of osteoarthritis. J Orthop Res Conf. 2017;35:e257

[29] Bossen D, Veenhof C, Van Beek KE, Spreeuwenberg PM, Dekker J, De Bakker DH (2013). Effectiveness of a web-based physical activity intervention in patients with knee and/or hip osteoarthritis: randomized controlled trial. J Med Internet Res.

[30] Wangberg SC, Bergmo TS, Johnsen JA (2008). Adherence in internet-based interventions. Patient Prefer Adherence.

[31] Kelders SM, Kok RN, Ossebaard HC, Van Gemert-Pijnen JE (2012). Persuasive system design does matter: a systematic review of adherence to web-based interventions. J Med Internet Res.

[32] Kloek CJ, Bossen D, De Bakker DH, Dekker J, Veenhof C (2016). Blended physical activity intervention with reduced face-to-face contact and usual physical therapy show similar effectiveness in patients with knee and hip osteoarthritis: a randomized controlled trial. Ann Rheum Dis.

[33] Veenhof C, Kloek C, Bossen D, Dekker J, De Bakker D (2015). The iterative development of e-exercise, a blended exercise intervention for patients with osteoarthritis of hip and/or knee. Ann Rheum Dis.

[34] Head KJ, Noar SM, Iannarino NT, Grant HN (2013). Efficacy of text messaging-based interventions for health promotion: a meta-analysis. Soc Sci Med (1982).

[35] Hall AK, Cole-Lewis H, Bernhardt JM (2015). Mobile text messaging for health: a systematic review of reviews. Annu Rev Public Health.

[36] Blake H, Roberts A, Batt M, Moses J (2015). Motive8!: feasibility of a text messaging intervention to promote physical activity in knee osteoarthritis. Int J Sports Exerc Med.

[37] Chen HC, Chuang TY, Lin PC, Lin YK, Chuang YH (2017). Effects of messages delivered by Mobile phone on increasing compliance with shoulder exercises among patients with a frozen shoulder. J Nurs Scholarsh.

[38] Muller AM, Khoo S, Morris T (2016). Text messaging for exercise promotion in older adults from an upper-middle-income country: randomized controlled trial. J Med Internet Res.

[39] de Jongh T, Gurol-Urganci I, Vodopivec-Jamsek V, Car J, Atun R (2012). Mobile phone messaging for facilitating self-management of long-term illnesses. Cochrane Database Syst Rev.

[40] Iribarren SJ, Cato K, Falzon L, Stone PW (2017). What is the economic evidence for mHealth? A systematic review of economic evaluations of mHealth solutions. PLoS One.

[41] Stephens J, Allen J (2013). Mobile phone interventions to increase physical activity and reduce weight: a systematic review. J Cardiovasc Nurs.

[42] Bennell KL, Nelligan R, Dobson F, Rini C, Keefe F, Kasza J (2017). Effectiveness of an internet-delivered exercise and pain-coping skills training intervention for persons with chronic knee pain: a randomized trial. Ann Intern Med.

[43] Michie S, Atkins L, West R (2014). The behaviour change wheel: a guide to designing interventions.

[44] Michie S, van Stralen MM, West R (2011). The behaviour change wheel: a new method for characterising and designing behaviour change interventions. Implement Sc.

[45] Cane J, O'Connor D, Michie S (2012). Validation of the theoretical domains framework for use in behaviour change and implementation research. Implement Sci.

[46] Michie S, Richardson M, Johnston M, Abraham C, Francis J, Hardeman W (2013). The behavior change technique taxonomy (v1) of 93 hierarchically clustered techniques: building an international consensus for the reporting of behavior change interventions. Ann Behav Med.

[47] Chan AW, Tetzlaff JM, Altman DG, Laupacis A, Gotzsche PC, Krle AJK (2015). SPIRIT 2013 statement: defining standard protocol items for clinical trials. Revista panamericana de salud publica. Pan Am J Public Health.

[48] Boutron I, Moher D, Altman DG, Schulz KF, Ravaud P (2008). Extending the CONSORT statement to randomized trials of nonpharmacologic treatment: explanation and elaboration. Ann Intern Med.

[49] Moher D, Hopewell S, Schulz KF, Montori V, Gotzsche PC, Devereaux PJ (2012). CONSORT 2010 explanation and elaboration: updated guidelines for reporting parallel group randomised trials. Int J Surg.

[50] Zwarenstein M, Treweek S, Gagnier JJ, Altman DG, Tunis S, Haynes B (2008). Improving the reporting of pragmatic trials: an extension of the CONSORT statement. BMJ.

[51] Eysenbach G (2011). CONSORT-EHEALTH: improving and standardizing evaluation reports of web-based and mobile health interventions. J Med Internet Res.

[52] HONcode© [February, 2018]. Available from: https://www.hon.ch/HONcode/

[53] Medicine. MPUSNLo. [February, 2018] How to write easy-to-read health materials. 2016. Available from: http://www.nlm.nih.gov/medlineplus/etr.html.

[54] Santos PJF, Daar DA, Badeau A, Leis A. Readability of online materials for Dupuytren’s contracture. J Hand Ther. 2017;11(1):97-106. 10.1016/j.jht.2017.07.005 28843342

[55] Saunders CH, Elwyn G, Kirkland K, Durand MA (2018). Serious choices: a protocol for an environmental scan of patient decision aids for seriously ill people at risk of death facing choices about life-sustaining treatments. Patient.

[56] Cheng C, Dunn M (2015). Health literacy and the internet: a study on the readability of Australian online health information. Aust N Z J Public Health.

[57] McInnes N, Haglund BJ (2011). Readability of online health information: implications for health literacy. Inform Health Soc Care.

[58] Bennell KL, Campbell PK, Egerton T, Metcalf B, Kasza J, Forbes A (2017). Telephone coaching to enhance a home-based physical activity program for knee osteoarthritis: a randomized clinical trial. Arthritis Care Res.

[59] Garber CE, Blissmer B, Deschenes MR, Franklin BA, Lamonte MJ, Lee IM (2011). American College of Sports Medicine position stand. Quantity and quality of exercise for developing and maintaining cardiorespiratory, musculoskeletal, and neuromotor fitness in apparently healthy adults: guidance for prescribing exercise. Med Sci Sports Exerc.

[60] American College of Sports Medicine position stand (2009). Progression models in resistance training for healthy adults. Med Sci Sports Exerc.

[61] Abroms LC, Whittaker R, Free C, Mendel Van Alstyne J, Schindler-Ruwisch JM (2015). Developing and pretesting a text messaging program for health behavior change: recommended steps. JMIR mHealth and uHealth.

[62] Hawker GA, Mian S, Kendzerska T, French M (2011). Measures of adult pain: visual analog scale for pain (VAS pain), numeric rating scale for pain (NRS pain), McGill pain questionnaire (MPQ), short-form McGill pain questionnaire (SF-MPQ), chronic pain grade scale (CPGS), short Form-36 bodily pain scale (SF-36 BPS), and measure of intermittent and constant osteoarthritis pain (ICOAP). Arthritis Care Res.

[63] Roos EM, Roos HP, Lohmander LS, Ekdahl C, Beynnon BD (1998). Knee injury and osteoarthritis outcome score (KOOS)--development of a self-administered outcome measure. J Orthop Sports Phys Ther.

[64] Bellamy N, Buchanan WW, Goldsmith CH, Campbell J, Stitt LW (1988). Validation study of WOMAC: a health status instrument for measuring clinically important patient relevant outcomes to antirheumatic drug therapy in patients with osteoarthritis of the hip or knee. J Rheumatol.

[65] Osborne RH, Hawthorne G, Lew EA, Gray LC (2003). Quality of life assessment in the community-dwelling elderly: validation of the assessment of quality of life (AQoL) instrument and comparison with the SF-36. J Clin Epidemiol.

[66] Washburn RA, Smith KW, Jette AM, Janney CA (1993). The physical activity scale for the elderly (PASE): development and evaluation. J Clin Epidemiol.

[67] Lorig K, Chastain RL, Ung E, Shoor S, Holman HR (1989). Development and evaluation of a scale to measure perceived self-efficacy in people with arthritis. Arthritis Rheum.

[68] ten Klooster PM, Drossaers-Bakker KW, Taal E, van de Laar MA (2006). Patient-perceived satisfactory improvement (PPSI): interpreting meaningful change in pain from the patient’s perspective. Pain.

[69] Newman-Beinart NA, Norton S, Dowling D, Gavriloff D, Vari C, Weinman JA (2017). The development and initial psychometric evaluation of a measure assessing adherence to prescribed exercise: the exercise adherence rating scale (EARS). Physiotherapy.

[70] Cohen J. Statistical power analysis for the behavioral sciences. Routledge; 1988.

[71] Bellamy N, Carette S, Ford P, Kean W, Lussier A, Wells G (1992). Osteoarthritis antirheumatic drug trials. III. Setting the delta for clinical trials--results of a consensus development (Delphi) exercise. J Rheumatol.

[72] Angst F, Aeschlimann A, Stucki G (2001). Smallest detectable and minimal clinically important differences of rehabilitation intervention with their implications for required sample sizes using WOMAC and SF-36 quality of life measurement instruments in patients with osteoarthritis of the lower extremities. Arthritis Rheum.

[73] Adult Pre-exercise Screening System (2012). Australia: E.S.S.

