# Supplementary material for: Effectiveness of internet-delivered education and home exercise supported by behaviour change SMS on pain and function for people with knee osteoarthritis: a randomised controlled trial protocol
Source: BMC Musculoskelet Disord. 2019 Jul 27;20:342. doi: 10.1186/s12891-019-2714-x (PMC6661079; doi:10.1186/s12891-019-2714-x)
Supplement: Supplementary file 1 — Exercise barriers and the BCTs which were converted into individual SMS to address each barrier. Demonstrates the application of the BCW framework using COM-B categories, TDF domains and intervention functions. (PDF 63 kb) [file 12891_2019_2714_MOESM1_ESM.pdf]

Barrier	COM-B category	TDF Domain	Relevant Intervention function	BCT used within the SMS intervention
1. Forgetfulness	Psychological capability	10. Memory, attention and decision processes	Training	8.3 Habit formation
			Environmental restructuring	7.1 Prompts/cues
			Enablement	3.1 Social support (unspecified)
				10.9 Self-reward
				1.2 Problem Solving
				1.4 Action Planning
2. Too tired	Psychological capability<br><br>Reflective motivation*	10. Memory, attention and decision processes<br><br>8. Intentions	Education*	5.1 Information about health consequences *
			Persuasion*	-
			Training	-
			Environmental restructuring	-
			Enablement	1.4 Action planning
				15.4 Self talk
				15.1 Verbal persuasion about capability
				1.2 Problem Solving
				8.7 Graded tasks
3. Knee pain limiting perceived ability to exercise	Reflective motivation	4. Beliefs about Capabilities	Education	5.1 Information about health consequences
			Persuasion	-
			Enablement	8.7 Graded tasks
				12.4 Distraction
				15.4 Self talk
				1.4 Action Planning
				1.2 Problem Solving
4. Concern exercise (causing) pain + Fear of damaging knee further	Reflective motivation	6. Beliefs about consequences	Persuasion	5.1 Information about health consequences
			Enablement	1.2 Problem Solving
				1.4 Action planning
5. Lack of improvement with exercises	Automatic motivation	7. Reinforcement	Training	2.4 Self-monitoring outcome(s) of behaviour
				2.2 Feedback on behaviour
				4.4 Behavioural experiments
				4.1 Instruction on how to perform the behaviour
			Environmental restructuring	-

6.<br>Lack of enjoyment in exercise +<br>Boredom with exercise	Automatic motivation	13. Emotion	Persuasion	5.1 Information about health consequences
			Enablement	12.4 Distraction
				15.4 Self talk
				10.9 Self-reward
				12.1 Restructuring the physical environment
				1.2 Problem Solving
				1.4 Action Planning
				3.1 Social support (unspecified)
7.<br>Conflict with routines +<br>Lack of time	Reflective motivation	11. Environmental context and resources	Training	4.1 Instruction on how to perform the behaviour
			Environmental restructuring	-
			Enablement	3.1 Social support – unspecified
				1.2 Problem solving
				1.4 Action Planning
				15.3 Focus on past success
				1.3 Goal setting (outcome)
8.<br>Family commitments +<br>Increased social strain +<br>Life events	Social Opportunity	12. Social influences	Environmental restructuring	-
			Enablement	1.4 Action Planning
				2.4 Self-monitoring of outcomes of behaviour
				15.1 Verbal persuasion about capability
				1.2 Problem solving
				15.4 Self talk
				15.3 Focus on past success
				1.1 Goal setting (behaviour)

BCT= Behaviour change technique

BCW=Behaviour Change Wheel

Dash (-) indicates no BCT messages used in final SMS library from this intervention function
