# Supplementary material for: Effectiveness of internet-delivered education and home exercise supported by behaviour change SMS on pain and function for people with knee osteoarthritis: a randomised controlled trial protocol
Source: BMC Musculoskelet Disord. 2019 Jul 27;20:342. doi: 10.1186/s12891-019-2714-x (PMC6661079; doi:10.1186/s12891-019-2714-x)
Supplement: Supplementary file 2 — Exercise facilitators and the BCTs which were converted into individual SMS to address each facilitator Demonstrates the application of the BCW framework using COM-B categories, TDF domains and intervention functions. (PDF 35 kb) [file 12891_2019_2714_MOESM2_ESM.pdf]

Facilitator	COM-B category	TDF domain	Relevant Intervention Function	BCT used within the SMS intervention
1. Accurate disease knowledge	Psychological capability	1. Knowledge	Education	5.1 Information about health consequences
2. Prioritising exercise	Psychological capability	14. Behavioural regulation	Education	5.1 Information about health consequences
			Training	4.1 Instruction on how to perform the behaviour
				10.9 Self reward
			Enablement	10.9 Self reward
3. Integrating exercise into daily tasks	Psychological capability	14. Behavioural regulation	Education	5.1 Information about health consequences
			Training	4.1 Instruction on how to perform the behaviour
				10.9 Self reward
			Enablement	10.9 Self reward
4. Belief that you are taking control of own disability	Psychological capability	4. Beliefs about Capabilities	Education	5.1 Information about health consequences
			Persuasion	5.1 Information about health consequences
				6.2 Social Comparison
5. Perceived benefits of exercising	Psychological capability	6. Beliefs about consequences	Education	5.1 Information about health consequences
			Persuasion	5.1 Information about health consequences
				6.2 Social Comparison
6. Belief that exercise is good for health	Psychological capability	6. Beliefs about consequences	Education	5.1 Information about health consequences
			Persuasion	5.1 Information about health consequences
				6.2 Social Comparison
7. Positive outcome expectations	Psychological capability	6. Beliefs about consequences	Education	5.1 Information about health consequences
			Persuasion	5.1 Information about health consequences
				6.2 Social Comparison
8. Long-term goals as well as short-term goals	Psychological capability	9. Goals	Education	5.1 Information about health consequences
			Persuasion	5.1 Information about health consequences
				6.2 Social Comparison

				Enablement	10.9 Self reward
9.	Receiving medical advice to exercise	Automatic motivation	7. Reinforcement	Training	4.1 Instruction on how to perform the behaviour
					10.9 Self reward
				Environmental restructuring	-

BCT= Behaviour change technique

BCW=Behaviour Change Wheel

Dash (-) indicates no BCT messages used in final SMS library from this intervention function
